# Anti-diabetic treatment regulates pro-fibrotic TGF-β serum levels in type 2 diabetics

**DOI:** 10.1186/1758-5996-5-48

**Published:** 2013-08-31

**Authors:** Stefan Pscherer, Thomas Freude, Thomas Forst, Andreas K Nussler, Karl F Braun, Sabrina Ehnert

**Affiliations:** 1 Klinikum Traunstein, Diabetes Department, Cuno-Niggl-Straße 3, Traunstein 83278, Germany; 2 BG Trauma Center Tübingen, Schnarrenbergstraße 95, Tübingen 72076, Germany; 3 Department of Endocrinology, ikfe Mainz/University Mainz, Parcusstraße 8, Mainz 55116, Germany; 4 Department of Trauma Surgery, Klinikum rechts der Isar, Technische Universität München, Ismaningerstraße 22, Munich 81675, Germany

**Keywords:** Diabetes mellitus type 2, TGF-β, Insulin, Proinsulin, Glucose

## Abstract

**Background:**

The single-center, open-label, four-arm, exploratory study investigates the relation of different anti-diabetics to serum levels of active TGF-β, a known pro-fibrotic stimulus, before and after a defined test meal.

**Findings:**

We investigated sera of patients with type 2 diabetes mellitus (T2DM) treated with metformin and sulfonylurea, insulin glargine or a DPP-4 inhibitor (DPP4i). Patients’ sera were analyzed before and 5 h after a defined test meal at intervals of 30 min.

The sulfonylurea/metformin group exhibited the highest basal levels of active TGF-β (31.50 ± 3.58 ng/ml). The glargine/metformin group had active TGF-β levels (24.98 ± 1.90 ng/ml) that were comparable to those of the healthy participants (22.12 ± 2.34 ng/ml). The lowest basal levels of active TGF-β were detected in the DPP-4i/metformin group (12.28 ± 0.84 ng/ml). Following the intake of a standardized meal, active TGF-β levels decreased (approx. 30%) in healthy subjects as well as in the sulfonylurea/metformin group and in the glargine/metformin group. After 5 h, the active TGF-β levels were normalized to basal levels. Active TGF-β levels in the DPP-4i/metformin group did not change significantly after the test meal. Overall plasma levels of insulin and proinsulin were comparable between healthy participants, and T2DM patients in the glargin/metformin group and in the DPP4i/metformin group. However, no correlation between active TGF-β levels, glucose, insulin or pro-insulin levels was detected.

**Conclusions:**

T2DM patients often exhibit elevated levels of pro-fibrotic active TGF-β. Our results suggest that glargine/metformin and DPP4i/metformin treatment may more effectively reduce active TGF-β serum levels than the sulfonylurea/metformin treatment.

## Background

Diabetes mellitus type 2 (T2DM) is a chronic metabolic disorder with a dramatically increasing prevalence [1]. T2DM stems from a progressive insulin secretory defect on the background of insulin resistance [2]. Prolonged insufficient metabolic control with constantly elevated HbA1c levels inevitably leads to complications, most commonly to diabetic nephropathy, diabetic retinopathy, diabetic neuropathy, and macrovascular problems. TGF-β_1_, a known pro-fibrotic factor, activates the production of extracellular matrix by mesangial cells and interstitial fibroblasts in the kidneys, and thus contributes to the manifestation of diabetic kidney disease [3]. In mesangial cells, it has been demonstrated that the expression and activity of TGF-β_1_ are directly influenced by glucose levels [4].

The aim of this study was investigating the influence of different pharmacological strategies in Type 2 diabetics on basal active TGF-β serum levels and active TGF-β serum levels after a standardized test meal in correlation with insulin, proinsulin and glucose levels as possible regulators.

### Study groups

The first publication of this single-center, open-label, four-arm, exploratory study investigated the influence of different pharmacological strategies on the postprandial processing of intact proinsulin in T2DM [5]. For the present study, each group consisted of 20 participants on average. Baseline demographics are shown in Table 1. In the control group, impaired glucose tolerance and diabetes mellitus were excluded by an oral glucose tolerance test. T2DM patients had been treated with a constant anti-diabetic dose according to the following pharmacological strategies for at least 6 months:

• sulfonylurea plus metformin (S+M) for 6 to 12 months

• the long-acting insulin analogue glargine plus metformin (G+M) for 6 to 12 months

• a DPP-4 inhibitor plus metformin (D+M) for the past 6 months with a stable anti-diabetic dosage during the past 3 months

**Table 1:** Study subjects

	**C**	**S + M**	**G + M**	**D + M**
**Participants**	20	21	20	19
**Ratio male/female**	12/8	16/5	15/5	11/8
**Age [a]**	65 ± 1.33	64 ± 1.5	65 ± 1.58	62 ± 1.92
**Duration of diabetes [a]**	-	7.9 ± 0.9	8.7 ± 1.3	6.8 ± 0.9
**BMI**	27.1 ± 3.5	31.6 ± 2.9	30.8 ± 2.9	30.2 ± 3.3
**HbA1c**	5.6 ± 0.3	7.0 ± 0.5	7.2 ± 0.2	6.6 ± 0.4
**Basal glucose [mg/dl]**	100.1 ± 1.5	148.0 ± 3.9***	146.0 ± 5.1***	144.3 ± 5.1***
**Basal insulin [pmol/l]**	9.02 ± 0.87	17.23 ± 1.63***	10.64 ± 1.59°°	11.70 ± 0.90°
**Basal proinsulin [pmol/l]**	5.26 ± 0.39	16.37 ± 1.45***	9.378 ± 0.97*^/^°°°	6.878 ± 0.49°°°

***p < 0.001 as compared to healthy controls.

°p < 0.05 °°p < 0.01 °°°p < 0.001 as compared to “S+M” group.

### Ethics statement

The study was performed according to the 1964 Declaration of Helsinki. Written informed consent was obtained from all patients. The study was approved by an independent ethics committee (Ethik-Kommission der Landesärztekammer Rheinland-Pfalz, Mainz, Germany) and a regulatory authority (The Bundesinstitut für Arzneimittel und Medizinprodukte, Bonn, Germany) [5].

### Serum and plasma analysis

Plasma levels of TGF-β, insulin, proinsulin, and blood glucose were measured after a fasting period and after a standardized meal for 5 hours. Plasma proinsulin and insulin levels were measured with an enzyme-linked immunosorbent assay [5]. Blood glucose levels were measured with an electrochemical biosensor [5]. Serum levels of active TGF-β were measured using TGF-β-reporter cells (MFB-F11) as reported [6,7].

### Statistics

Statistics and data calculation were performed with the GraphPad Prism software (La Jolla, USA). Results are expressed as mean ± SEM. Sample distribution was analyzed with the D’Agostino & Pearson omnibus normality test. Data sets were compared by one-way analysis of variance followed by Bonferroni’s multiple comparison test. *p* < 0.05 was taken as the minimum level of significance.

## Results

**Figure 1:**
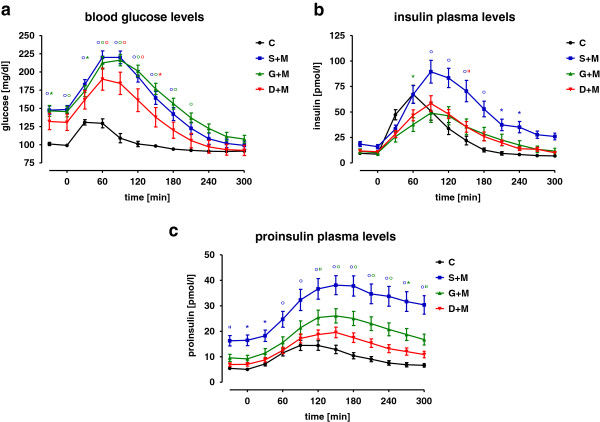
**Blood glucose, insulin and proinsulin levels after a test meal.** T2DM patients treated with metformin and sulfonylurea (S+M), insulin glargine (G+M) or a DPP-4 inhibitor (D+M) as well as non-diabetic controls (C) received a standardized test meal. Over the course of 5 h, **(a)** blood glucose, **(b)** insulin, and **(c)** proinsulin levels were determined at intervals of 30 min. Each group consisted of 20 individuals on average. All three serum parameters increased after the test meal reaching their peak between 60 and 90 min after the test meal. “ p < 0.05, * p < 0.01 and ° p < 0.001 as compared to C.

Lowest basal (fasting) blood glucose levels were detected in the control group. In all groups, blood glucose levels increased after the standardized test meal. The peak was reached after 30 to 60 min in the control group (~25% increase), and after 60 to 90 min in the T2DM groups (~50% increase). Blood glucose levels were normalized to baseline levels faster in the control group (2 h) than in all three T2DM groups (5 h) (Figure 1a).

Insulin plasma levels increased after the standardized test meal. The peak was reached after 30–60 min in the control group (~7-fold), and after 60–90 min in the T2DM groups (5 to 6-fold). Plasma insulin levels were normalized to baseline levels faster in the control group (after 3 h) than in all T2DM groups (after 5 h) (Figure 1b).

The lowest basal plasma proinsulin levels were observed in the control group, while the highest levels were found in the T2DM patients treated with S+M. In all groups, plasma proinsulin levels increased after the standardized test meal. In the control group, the peak was reached after 90–120 min (~3-fold increase), which decreased to baseline levels after 5 h. In all three T2DM groups, peak plasma proinsulin levels were observed after 150 min. Plasma proinsulin levels in these groups did not normalize to baseline levels within the observed time frame (Figure 1c).

**Figure 2:**
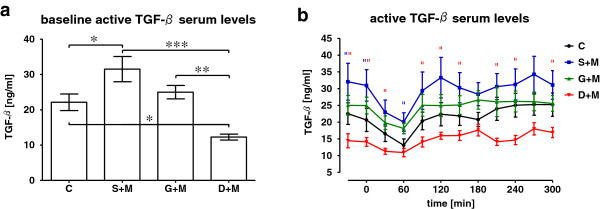
**Active TGF-β serum levels before and after a test meal.** T2DM patients treated with metformin and sulfonylurea (S+M), insulin glargine (G+M) or a DPP-4 inhibitor (D+M) as well as non-diabetic controls (C) received a standardized test meal. Over the course of 5 h, active TGF-β serum levels were measured at 30 min intervals. Each group consisted of 20 individuals on average. **(a)** Basal active TGF-β serum levels strongly depend on the pharmaceutical strategy. * p < 0.05, ** p < 0.01 and *** p < 0.001. **(b)** After the test meal, active TGF-β serum levels dropped after having reached their peak about 60 min after the test meal and then normalized to baseline levels. “ p < 0.05 as compared to C.

Healthy controls (22.12±2.34 ng/ml) and T2DM patients treated with G+M (24.98±1.90 ng/ml) had comparable basal (fasting) serum levels of active TGF-β. While T2DM patients receiving S+M (31.50±3.58 ng/ml) had significantly higher baseline (fasting) active TGF-β serum levels, T2DM patients treated with D+M exhibited significantly lower fasting active TGF-β serum levels (12.28±0.84 ng/ml) (Figure 2a). Following a standardized meal, the active TGF-β serum levels strongly decreased in the control group and in the T2DM patients treated with S+M or G+M. In all three groups, active TGF-β serum levels dropped in the first 60 min after the test meal, and reached the initial baseline levels at the end of the observation period. The active TGF-β serum levels of T2DM patients treated with D+M did not vary significantly throughout the observation period (Figure 2b).

**Figure 3:**
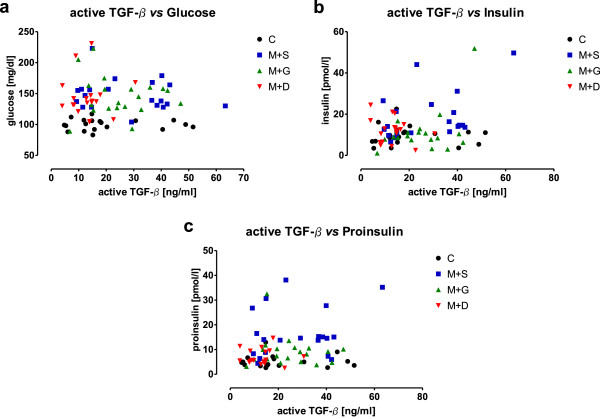
**Correlation analysis between basal active TGF-β serum levels and fasting blood glucose, insulin and proinsulin levels.** Basal active TGF-β serum levels of T2DM patients treated with metformin and sulfonylurea (S+M), insulin glargine (G+M) or a DPP-4 inhibitor (D+M) as well as of non-diabetic controls (C) were correlated with basal **(a)** blood glucose levels, **(b)** insulin levels, and **(c)** proinsulin levels.

To determine the total amount of active TGF-β over the entire observation time, the area under curve (AUC) was defined. The AUCs for active TGF-β, glucose, insulin and pro-insulin are presented in Table 2. However, no correlation between active TGF-β and basal glucose, insulin or pro-insulin levels was observed (Figure 3a-c).

**Table 2:** Area under the curve (AUC) from −30 to 300 min

**Area under the curve**	**C**	**S + M**	**G + M**	**D + M**
**TGF-β [ng/ml]**	7014	9689	8047	4873
**Glucose [mg/dl]**	33961	51821	53402	44817
**Insulin [pmol/l]**	8268	16057	8922	9493
**Proinsulin [pmol/l]**	3165	9832	6294	4523

## Discussion

TGF-β is involved in many cellular processes, e.g. the modulation of the immune system and enhanced formation of extracellular matrix [8,9]. Increased matrix formation is one reason why TGF-β is regarded as a key player in the development of fibrosis in many organs [10]. Especially in the kidneys, excessive matrix formation by mesangial cells and interstitial fibroblasts contributes to the manifestation of diabetic kidney disease [3]. A reduced activation of TGF-β in the kidney may reduce the risk of development of fibrosis. In the present study, we analyzed serum levels of active TGF-β in patients with T2DM treated according to different anti-diabetic therapies. So far, little is known about the effect of different blood glucose-lowering interventions on TGF-β. The fasting active TGF-β serum levels were significantly higher in T2DM patients treated with S+M in comparison to the three other groups. The fasting active TGF-β serum levels of T2DM patients treated with G+M were similar to those of the non-diabetic controls. T2DM patients treated with D+M even had significantly decreased fasting active TGF-β serum levels comparable to those of the non-diabetic controls. After the test meal, the active TGF-β serum levels dropped for around one hour and increased again to baseline levels during the following four hours. This decrease of TGF-β after the meal suggests a direct connection to insulin secretion. However, we were not able to show a direct correlation between these two factors, possibly due to a timely shift in the response or the involvement of more factors. As the insulin secretion occurs in a physiologically biphasic and pulsatile manner [11], our T2DM patients already exhibited a delayed and prolonged insulin secretion when T2DM was diagnosed.

The three T2DM patient groups were commonly treated with the biguanide metformin. As we observed significantly different fasting active TGF-β serum levels between the individual treatment groups, we suppose that metformin does not directly affect the expression and activation of TGF-β. This is supported by data demonstrating that the treatment of mesangial cells with metformin does not influence the glucose transport or the expression of fibronectin and collagen at physiological conditions [12].

The sulfonylurea tolazamide significantly stimulates the secretion of TGF-β_1_, and thus the synthesis and accumulation of collagen and fibronectin in mesangial cells [12]. This supports our findings that T2DM patients treated with S+M exhibit increased fasting active TGF-β serum levels. However, it has been discussed recently that this observation might be strongly glucose-dependent, as mesangial cells exposed to different concentrations of glucose and to the sulfonylurea glibenclamide exhibited an increased expression of TGF-β only at high glucose concentrations [4,13,14]. In this setting, a 10-fold concentration of glibenclamide even inhibited collagen synthesis [13,14].

In our study, T2DM patients receiving D+M had the lowest fasting active TGF-β serum levels. This is supported by a study with 10-week-old db/db mice, where the treatment with a DPP-4 inhibitor extenuated the expression of TGF-β_1_ and reduced myocardial fibrosis [15]. In streptozotocin-induced diabetic rats, 24 weeks of treatment with a DPP-4 inhibitor reduced the TGF-β_1_ expression. Thus, this inhibitor was reno-protective [16]. Remarkably, fasting active TGF-β serum levels in this group were significantly lower compared to the non-diabetic controls. For many bodily functions, a fine adjustment of TGF-β expression and activation is necessary. A complete abortion of the TGF-β activation, as observed in our T2DM patients treated with D+M after the test meal, might be critical. Nevertheless, the role of TGF-β in T2DM must be investigated further, since it seems to suppress atherosclerosis in diabetic patients [17].

## Conclusions

Our study suggests that different blood glucose-lowering pharmacological interventions have a significant impact on the serum levels of pro-fibrotic active TGF-β. Treatment with M+G as well as with M+D was superior to the treatment with M+S in keeping low fasting active TGF-β serum levels. However, further investigations will be needed to clarify the clinical significance of TGF-β regarding the development of kidney fibrosis and its role in suppressing atherosclerosis or other pathologies in patients with T2DM.

## Abbreviations

C: Non-diabetic controls; M+D: Treatment with metformin plus a DPP4 inhibitor; M+G: Treatment with metformin plus insulin analogue glargine; M+S: Treatment with metformin plus sulfonylurea; T2DM: Type 2 Diabetes mellitus.

## Competing interests

The authors declare that there do not exist any competing interests regarding this paper.

## Authors’ contributions

SP, AKN and SE conceived and designed the study. TFo acquired the samples. SP and KFB performed the trials. SP, TFr, AKN and SE analyzed the data. SP and SE wrote the manuscript. TFr, TFo, AKN and KFB revised the manuscript critically. All authors have read and approved the final version of the manuscript.
